# Elevated S100A8 in nasal mucosa correlates with epithelial barrier dysfunction in allergic rhinitis

**DOI:** 10.1016/j.bjorl.2026.101800

**Published:** 2026-03-19

**Authors:** Xiaocong Deng, Di Wu, Zhenlin Cai, Yong Qian

**Affiliations:** Hainan Cancer Hospital, Department of Head and Neck Surgery, Haikou, Hainan, China

**Keywords:** Allergic rhinitis, S100A8, Epithelial barrier, Tight junction

## Abstract

**Objective:**

Epithelial barrier dysfunction is central to the pathogenesis of Allergic Rhinitis (AR), yet its molecular mechanisms remain unclear. S100A8 is implicated in epithelial barrier disruption, but its role in AR is not fully understood. This study aimed to investigate S100A8 expression in AR and its association with barrier dysfunction.

**Methods:**

Nasal mucosal tissues were collected from 30 AR patients and 30 Healthy Controls (HCs). Expression levels of S100A8 and tight junction proteins (ZO-1, E-cadherin, occludin) were assessed by Immunofluorescence (IF), Western Blot (WB), and RT-qPCR. Correlation analysis was performed to evaluate the relationship between S100A8 and tight junction markers. Human Nasal Epithelial Cells (HNECs) from HCs were stimulated with House Dust Mite (HDM) extracts, and S100A8 expression was silenced using siRNA to assess its role in HDM-induced barrier disruption.

**Results:**

WB and RT-qPCR results showed that the expression of S100A8 was significantly enhanced in nasal mucosal samples of AR patients compared to the HC group. S100A8 mRNA level was significantly elevated, and its level was positively correlated with the Visual Analog Scores (VAS) and total nasal symptom scores (TNSS) of the patients. Moreover, IF further confirmed that S100A8 was enhanced in the AR group, and mainly localised in the nasal epithelium. The expression of ZO-1, E-cadherin, and occludin was markedly reduced in AR patients and showed a negative correlation with S100A8 levels. In vitro, HDM stimulation of HNECs led to a dose- and time-dependent increase in S100A8 expression, along with reduced levels of tight junction proteins. Silencing S100A8 via siRNA significantly restored tight junction protein expression, indicating that S100A8 inhibition may help preserve nasal epithelial barrier integrity.

**Conclusion:**

S100A8 is upregulated in AR and associated with disease severity and epithelial barrier dysfunction. Targeting S100A8 may offer a therapeutic strategy to preserve nasal barrier integrity in AR.

**Level of evidence:**

Level 3.

## Highlights

• S100A8 is upregulated in AR and correlates with disease severity.
• S100A8 negatively regulates tight junction proteins in the nasal epithelium.
• Knocking down S100A8 restores the barrier integrity in HDM-exposed HNECs.

## Introduction

Allergic Rhinitis (AR) is a Th2-mediated chronic inflammatory disease of the upper respiratory tract, and eosinophilic infiltration of the nasal mucosa is a hallmark feature.^1^^,^^2^ Epidemiological studies indicate that AR affects approximately 10 %–20 % of the global population and seriously affects people's quality of life, including learning and daily activities.^3^^,^^4^ Recently, there has been a keen academic interest in exploring the pathogenic mechanisms of AR, in which nasal epithelial barrier dysfunction has emerged as a focus.^5^ The nasal epithelium is the front-line barrier to inhaled allergens and pathogens and is essential for maintaining mucosal immune homeostasis.^6^^,^^7^ However, epithelial integrity is often compromised in patients with AR, leading to increased permeability that promotes allergen penetration, antigen presentation, and sustained immune activation.^8^^,^^9^ Although barrier dysfunction is increasingly recognized as a pathogenesis of AR, the underlying molecular mechanisms leading to epithelial disruption are still not fully understood.

S100A8, a calcium-binding protein of the S100 family, is increasingly recognized for its involvement in mucosal inflammation and epithelial damage.^10^, ^11^, ^12^ S100A8 plays an important role in the development of inflammatory diseases by amplifying the inflammatory response through binding to related receptors.^13^, ^14^, ^15^ Previous studies have found that elevated S100A8 expression is observed in chronic inflammatory diseases, including asthma and inflammatory bowel disease, and that it contributes to epithelial barrier damage and disease progression.^16^^,^^17^ Our previous study found that serum and tissue levels of S100A8 were significantly elevated in patients with recurrent Chronic Rhinosinusitis with Nasal Polyp (CRSwNP), and S100A8 appears to be a potential objective biomarker for predicting CRSwNP recurrence.^18^ However, the specific role of S100A8 in AR, especially in relation to nasal epithelial barrier dysfunction, remains largely unexplored. Therefore, studying S100A8 in AR may provide new insights into the pathogenesis of the disease and identify potential therapeutic targets to protect mucosal barrier function.

## Methods

### Study subjects and sample collection

All participants provided informed consent, and the study was approved by the Institutional Ethics Committee of Hainan Cancer Hospital. A total of 30 patients diagnosed with Allergic Rhinitis (AR) and 30 Healthy Controls (HCs) were enrolled from Hainan Cancer Hospital between January 2024 and June 2024. AR diagnosis was based on clinical symptoms and positive serum-specific IgE or skin prick test against House Dust Mite (HDM).^19^ Individuals with respiratory infections, autoimmune diseases, or other chronic inflammatory disorders were excluded. Peripheral blood samples were collected for eosinophil count analysis. Nasal mucosal tissues were obtained from AR patients undergoing inferior turbinate surgery. The HCs group comprised individuals undergoing surgery for structural nasal septal deviation, with no history of allergic diseases, no symptoms of allergic rhinitis, and negative serum total IgE and dust mite-specific IgE tests. The AR group and HC group showed no statistically significant differences in basic characteristics such as age and gender (Table 1).

**Table 1:** Demographic and clinical characteristics of AR patients and HC.

Variable	HC (n = 30)	AR (n = 30)	p
Gender, male/female (n)	17/13	20/10	0.426
Age, year	40.0 ± 11.6	33.6 ± 9.8	0.230
Smoking, Yes/No	8/22	7/23	0.766
Drinking, Yes/No	9/21	11/19	0.584
Blood eosinophil counts, 10^9^/L	0.2 ± 0.1	0.4 ± 0.2	<0.001
Blood eosinophil percentage, %	2.5 ± 1.0	5.0 ± 2.1	<0.001

HC, Healthy Control; AR, Allergic Rhinitis.

### Western Blot (WB) analysis

Total protein from nasal tissues and Human Nasal Epithelial Cells (HNECs) was extracted using RIPA buffer supplemented with protease inhibitors. Protein concentration was measured by the BCA assay. Equal amounts of protein were separated by SDS-PAGE and transferred onto PVDF membranes. After blocking with 5% skim milk, membranes were incubated with primary antibodies against S100A8 (1:500, Affinity), ZO-1 (1:500, Affinity), E-cadherin (E-cad, 1:500, Affinity), and occludin (1:500, Affinity) overnight at 4 °C, followed by HRP-conjugated secondary antibodies. Bands were visualized using Enhanced Chemiluminescence (ECL) and quantified using ImageJ software.

### Quantitative real-time PCR (RT-qPCR)

Total RNA was extracted from nasal tissues using TRIzol reagent (Invitrogen) and reverse-transcribed into cDNA using the PrimeScript RT reagent kit (Takara). RT-qPCR was carried out on an ABI PRISM 7300 Detection System (Applied Biosystems, Waltham, USA) with SYBR Premix EX Taq (Servicebio, Wuhan, China). Relative gene expression levels were normalized to GAPDH. Primer sequences are listed in Supplementary Table S1.

### Immunofluorescence staining (IF)

Inferior turbinate tissue specimens were fixed in 10% neutral-buffered formalin at room temperature for 24 h. Following fixation, samples were washed in Phosphate-Buffered Saline (PBS) and processed using standard paraffin-embedding protocols. Briefly, tissues were dehydrated through a graded ethanol series (70%, 80%, 95%, and 100%, 1 h), cleared in xylene (two changes, 30 min), and infiltrated with melted paraffin wax (three changes, 60 min each at 60 °C). Samples were then embedded in paraffin blocks and sectioned at 4 μm thickness for subsequent immunofluorescence staining. After antigen retrieval and blocking, the sections were incubated overnight at 4 °C with primary antibodies against S100A8 (1:200, Affinity), ZO-1 (1:200, Affinity), E-cad (1:200, Affinity), and occludin (1:200, Affinity). After washing, appropriate fluorescent secondary antibodies were applied for 1 h at room temperature. Nuclei were counterstained with DAPI, and images were captured using a fluorescence microscope.

### Primary HNECs culture and house dust mite (HDM) stimulation

Primary HNECs were isolated from the nasal mucosa of HCs and cultured in PneumaCult™-Ex Plus Medium (Stemcell Technologies Inc., Vancouver, Canada) at 37 °C with 5% CO₂ for further expansion. Subsequently, these cultured cells were identified via immunofluorescence. The result demonstrated that these cultured cells expressed the epithelial cell marker Pan-Cytokeratin (1:200, Proteintech) but did not express the fibroblast marker α-SMA (1:200, Affinity), confirming that these cells obtained were pure HNECs and effectively ruling out the possibility of fibroblast contamination (Supplementary Fig. S1). When the cells reach 80% confluence, they are digested and seeded into a 6-well plate, and the cells designated for fluorescence detection are cultured on a coverslip. Upon reaching 70% confluence, cells were treated with different concentrations of HDM (Baar, Switzerland) extract (0–100 μg/mL) for 24 h. For S100A8 knockdown, HNECs were transfected with S100A8-specific siRNA (si-S100A8,20 nM) or scrambled siRNA (si-NC,20 nM) using Lipofectamine 3000 (Invitrogen). After 48 h of transfection, cells were stimulated with HDM extract for subsequent analysis.

### Statistical analysis

All statistical analyses were performed using GraphPad Prism 9.0. Continuous variables were firstly assessed for normality via the Shapiro–Wilk test. Data meeting normality requirements were expressed as mean ± standard deviation, with intergroup comparisons conducted using independent samples *t*-tests; data not meeting normality requirements were expressed as median (interquartile range), with intergroup comparisons conducted using the Mann–Whitney *U* test. Categorical variables were described by frequency, with intergroup comparisons performed using chi-square tests. Correlation analysis employed Pearson's correlation coefficient for normally distributed data and Spearman's correlation coefficient for non-normally distributed data, based on data distribution characteristics. For multiple group comparisons, under the assumptions of normal distribution and homogeneity of variance, one-way analysis of variance (ANOVA) is employed. If the results are statistically significant, Tukey's multiple comparisons test is further applied for pairwise post-hoc comparisons. A p-value <0.05 was considered statistically significant.

## Results

### Clinical characteristics of enrolled subjects

This study included 30 patients with AR and 30 Healthy Controls (HCs). Clinical characteristics are summarized in Table 1. The AR group showed significantly elevated peripheral blood eosinophil counts and percentages compared to the HC group, while no significant differences were observed in gender, age, BMI, smoking, or alcohol consumption between the groups.

### Elevated S100A8 expression correlates with AR disease severity

**Fig. 1:**
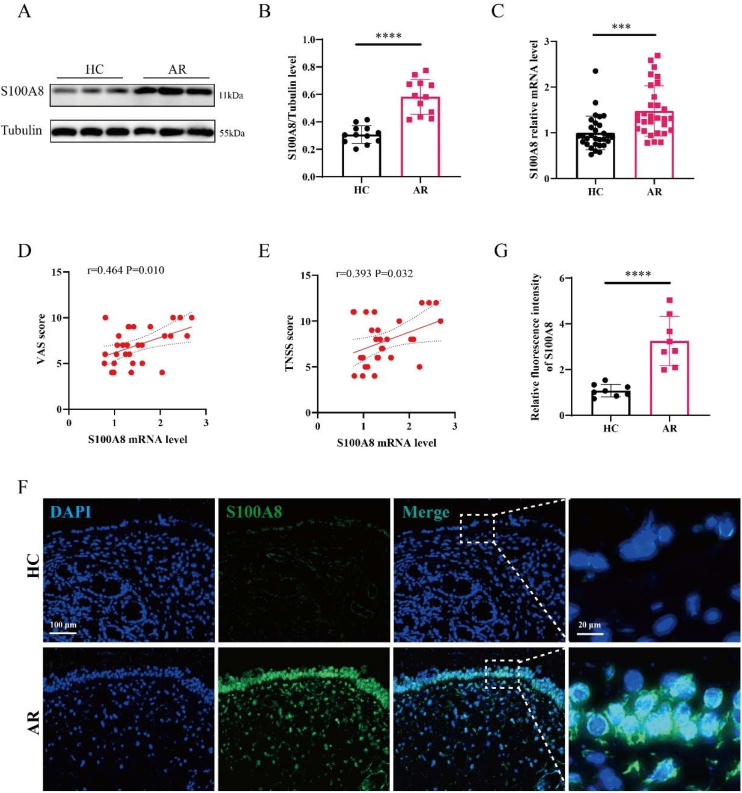
Increased expression of S100A8 in AR tissues. (A–B) WB showing tissue S100A8 protein expression (n = 12); (C) RT-qPCR analysis of tissue S100A8 (n = 30); (D–E) The correlation between S100A8 mRNA expression and VAS and TNSS score in AR patients; (F–G) IF staining for S100A8 between the two groups. Statistical analysis using t-test. Correlation analysis using Pearson's correlation coefficient. AR, Allergic Rhinitis; HC, Healthy Control; IF, Immunofluorescence; WB, Western Blotting; VAS, Visual Analog Scale; TNSS, Total Nasal Symptom Score. *** p < 0.001, **** p < 0.0001.

As shown in Fig. 1A–B, WB analysis confirmed significantly elevated S100A8 protein levels in the AR group. Consistently, RT-qPCR revealed a robust increase in S100A8 mRNA expression in AR patients compared to HCs (Fig. 1C). Moreover, correlation analysis demonstrated that elevated S100A8 mRNA levels were positively associated with both VAS scores (*r* = 0.464, p = 0.010) and TNSS scores (*r* = 0.393, p = 0.032) (Fig. 1D‒E), indicating a strong link between S100A8 expression and clinical disease severity. IF staining results showed that compared with HCs, S100A8 expression was markedly upregulated in nasal mucosal tissues from AR patients and was predominantly localized to the epithelium (Fig. 1F‒G). These findings suggest that S100A8 may play a pivotal role in the pathogenesis and progression of AR, and could serve as a potential biomarker reflecting disease activity.

### Upregulation of S100A8 is associated with epithelial barrier dysfunction in AR

**Fig. 2:**
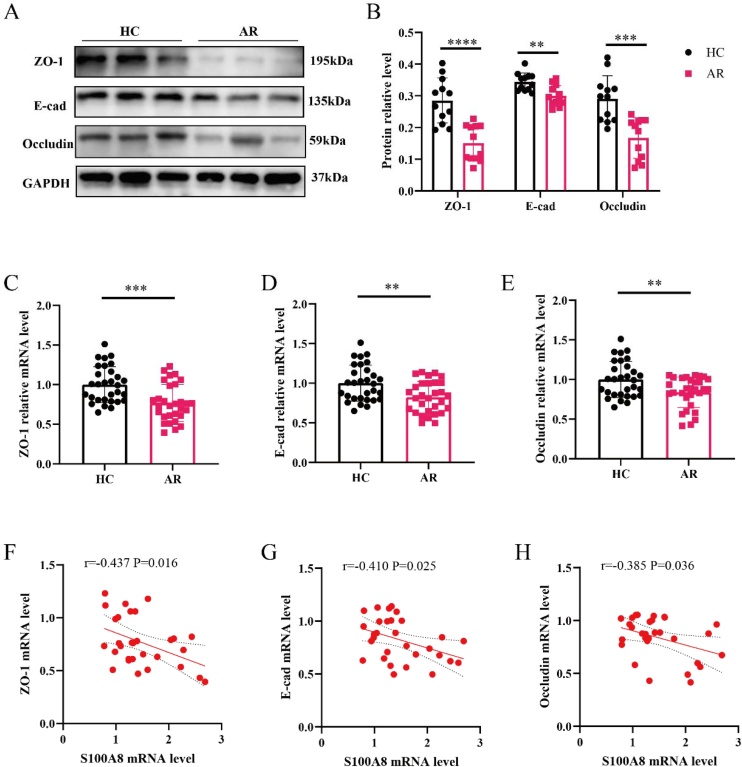
Increased S100A8 is associated with epithelial tight junction markers expression in AR patients. (A–B) WB for tight junction markers (n = 12); (C–E) RT-qPCR for tight junction markers between the two groups (n = 30); (F–H) The correlations between S100A8 mRNA and tight junction markers mRNA in AR patients (n = 30). Statistical analysis using t-test. Correlation analysis using Pearson's correlation coefficient. AR, Allergic Rhinitis; HC, Healthy Control; E-cad, E-Cadherin. ** p < 0.01, *** p < 0.001.

Previous studies have highlighted epithelial barrier dysfunction as a key pathological feature of the mucosa in AR.^5^^,^^8^ However, whether elevated expression of S100A8 contributes to AR pathogenesis by impairing nasal epithelial cell function and disrupting mucosal barrier integrity remains to be fully elucidated. In this study, WB staining in Fig. 2A–B revealed a notable reduction in protein expression of tight junction markers in nasal tissues from AR patients compared to HCs. To further assess barrier-related gene expression, RT-qPCR analysis found that the expression levels of ZO-1, E-cad and occludin were all significantly downregulated in the AR group (Fig. 2C‒E). Importantly, correlation analysis demonstrated that S100A8 mRNA levels in AR nasal mucosa were negatively associated with the expression of these tight junction components (Fig. 2F‒H). Collectively, these findings suggest that aberrant overexpression of S100A8 may compromise epithelial barrier integrity by downregulating tight junction proteins, leading to mucosal dysfunction in AR.

### S100A8 is involved in HDM-induced epithelial tight junction disruption

**Fig. 3:**
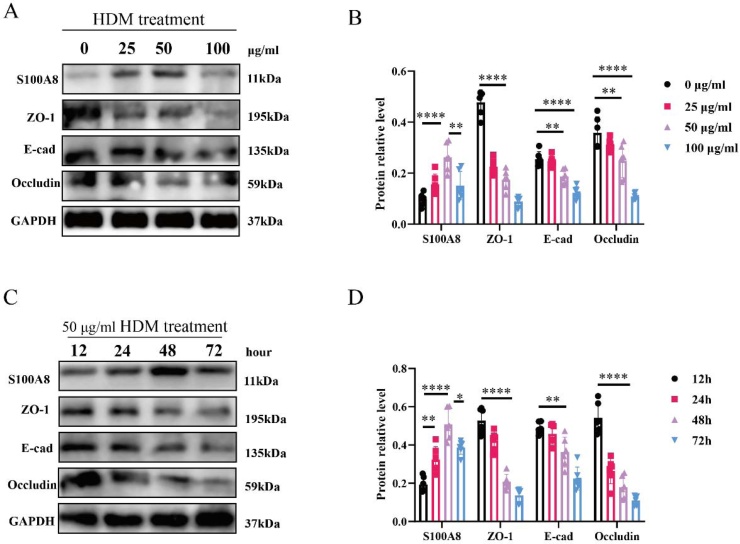
HDM stimulation promotes S100A8 expression and inhibits tight junction marker expression in HNECs. (A) Different HDM concentrations for intervening HNECs; (B) Different time of intervening HNECs; Statistical analysis using Tukey's multiple comparisons test. HDM, House Dust Mite; HNECs, Human Nasal Epithelial Cells. * p < 0.05, ** p < 0.01, **** p < 0.0001.

**Fig. 4:**
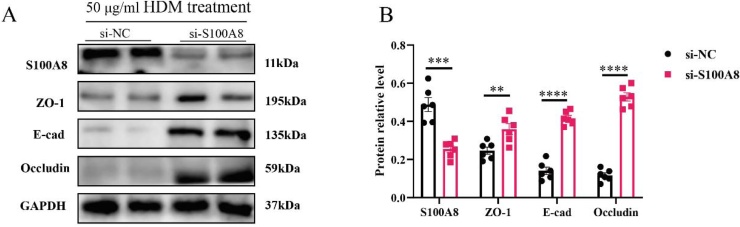
Inhibiting S100A8 alleviates the suppression of tight junction marker expression in HNECs mediated by HDM. (A-B) WB analysis of tight junction markers expression after si-S100A8 intervention. Statistical analysis using t-test. WB, western blotting; HDM, house dust mite. ** P < 0.01, *** P < 0.001, **** P < 0.0001.

To further investigate the role of S100A8 in epithelial barrier integrity, HNECs were isolated from HCs and exposed to HDM extract. WB analysis demonstrated that S100A8 expression increased progressively with HDM concentrations up to 50 μg/mL, accompanied by a dose-dependent reduction in the tight junction proteins ZO-1, E-cadherin, and occludin (Fig. 3A). However, at 100 μg/mL, S100A8 expression declined markedly, suggesting a potential cytotoxic effect at this high dose. Based on these findings, 50 μg/mL was selected for subsequent time-course experiments, which was consistent with previous studies.^20^ Next, we evaluated the impact of exposure duration at this optimized concentration. S100A8 expression and tight junction protein loss were evident as early as 24 h and peaked at 48 h, whereas prolonged exposure to 72 h resulted in a reduction of S100A8 levels (Fig. 3B–D), again indicative of possible cytotoxicity. Therefore, subsequent experiments were conducted under optimized conditions of 50 μg/mL HDM for 48 h. Notably, WB results further showed that siRNA-S100A8 effectively inhibited HDM-induced downregulation of ZO-1, E-cad and occluding (Fig. 4). Collectively, these findings indicate that S100A8 plays a pivotal role in mediating HDM-induced disruption of epithelial tight junctions, highlighting its potential as a therapeutic target for epithelial barrier protection in allergic rhinitis.

## Discussion

In this study, we investigated the expression and functional role of S100A8 in the nasal mucosa of patients with AR. Through a combination of clinical sample analysis and in vitro experiments, we found that S100A8 was significantly upregulated in AR, mainly localized in the nasal mucosal epithelium, and the elevated S100A8 was positively correlated with the disease severity. Furthermore, high expression of S100A8 was associated with barrier dysfunction, suggesting a potential role for S100A8 in the pathogenesis and progression of AR.

S100A8, a calcium-binding protein of the S100 family, has been increasingly implicated in inflammatory processes and epithelial dysfunction across various airway diseases.^21^ While primarily known for its role in innate immunity and neutrophil chemotaxis,^22^^,^^23^ emerging evidence suggests that S100A8 might directly modulate epithelial cell behavior and junctional stability.^24^^,^^25^ Nakatani et al.^25^ found that S100A8 caused nasal epithelial cells to produce interleukin-1β, which may be related to the pathogenesis of eosinophilic chronic rhinosinusitis. In this study, we identified a significant upregulation of S100A8 expression in the nasal mucosa of AR patients compared to controls. Importantly, correlation analyses revealed that S100A8 mRNA levels were positively associated with both TNSS and VAS scores, indicating a potential role for S100A8 in disease severity and progression. These findings indicated that S100A8 might be actively involved in the pathophysiological process of AR, but the specific role is unclear.

Epithelial barrier dysfunction has long been recognized as a key histopathological feature of AR, rendering the nasal mucosa more permeable to allergens, pathogens, and environmental irritants, thereby exacerbating inflammation and contributing to chronic disease progression.^26^, ^27^, ^28^, ^29^ Recent advances in molecular profiling have emphasized the complex cellular and molecular changes occurring within epithelial compartments during allergic inflammation.^30^^,^^31^ In our study, patients with AR exhibited a marked downregulation of critical tight junction proteins, including ZO-1, E-cad, and occludin, indicative of compromised epithelial integrity. Moreover, their expression levels were inversely correlated with S100A8, implying that S100A8 overexpression might contribute to epithelial barrier disruption. These observations align with prior findings where barrier defects were associated with enhanced allergic sensitization and inflammatory responses in AR and other allergic airway diseases.^32^^,^^33^

To further test this hypothesis, we established an in vitro model using HNECs treated with HDM. HDM stimulation resulted in a dose-dependent increase in S100A8 expression along with a parallel reduction in tight junction proteins, confirming that S100A8 is inducible under allergenic stimulation and may be involved in barrier damage. Importantly, siRNA-S100A8 markedly attenuated the HDM-induced downregulation of ZO-1, E-cad, and occludin, and preserved tight junction integrity, as confirmed by both IF and WB analyses. These results provide strong experimental evidence that S100A8 mediates allergen-induced epithelial dysfunction, potentially acting as an upstream effector of barrier damage in AR.

However, several limitations must be acknowledged. First, the sample size was limited, and the cohort was recruited from a single institution, which may reduce the generalizability of our findings. Second, although we demonstrated the involvement of S100A8 in HDM-induced epithelial dysfunction in vitro, more detailed mechanistic studies, such as those involving downstream signaling pathway analyses are required. Finally, in vivo validation using animal models of AR would strengthen the translational relevance of our findings.

## Conclusion

Our study has identified S100A8 as a critical mediator of epithelial barrier dysfunction in AR. By elucidating the link between S100A8 overexpression and tight junction disruption, we provide novel insights into AR pathogenesis and offer a potential therapeutic target for maintaining epithelial integrity in allergic airway diseases.

## ORCID ID

Xiaocong Deng: 0000-0001-7439-2009

Di Wu: 0009-0000-2120-0472

Zhenlin Cai: 0000-0002-5266-4920

## Funding

This research did not receive any specific grant from funding agencies in the public, commercial, or not-for-profit sectors.

## Data availability statement

The authors declare that all data are available in repository.

## Declaration of competing interest

The authors declare no have conflicts of interest.
