# Supplementary material for: Elevated S100A8 in nasal mucosa correlates with epithelial barrier dysfunction in allergic rhinitis
Source: Braz J Otorhinolaryngol. 2026 Mar 19;92(3):101800. doi: 10.1016/j.bjorl.2026.101800 (PMC13019065; doi:10.1016/j.bjorl.2026.101800)

BJORL-D-25-00183_Supplementary material

**Table S1** Primer sequence.

| **Gene** | **Forward primer** | **Reverse primer** |
| --- | --- | --- |
| GAPDH | TGCACCACCAACTGCTTAGCACCCC | AGCCTCAGTCCCATTCCCCAGCTCT |
| ZO-1 | TGACTGGTTTCGCCGAGAC | CGCACACCGTGATACCAATG |
| E-cadherin | CTCAAACGACACCCCTTGGA | CCCACCAGGTTTTTGCAGTC |
| Occludin | ACTTGTGCTGAAGAATTTTAGGACA | GCACATATAGCCGCACTAGC |

**Figure S1** Immunofluorescence staining identified HNECs. Immunofluorescence results demonstrated that the cells expressed the basal epithelial cell marker pan-cytokeratin but did not express the fibroblast marker α-SMA. HNECs, human nasal epithelial cells; α-SMA, Alpha-Smooth Muscle Actin.


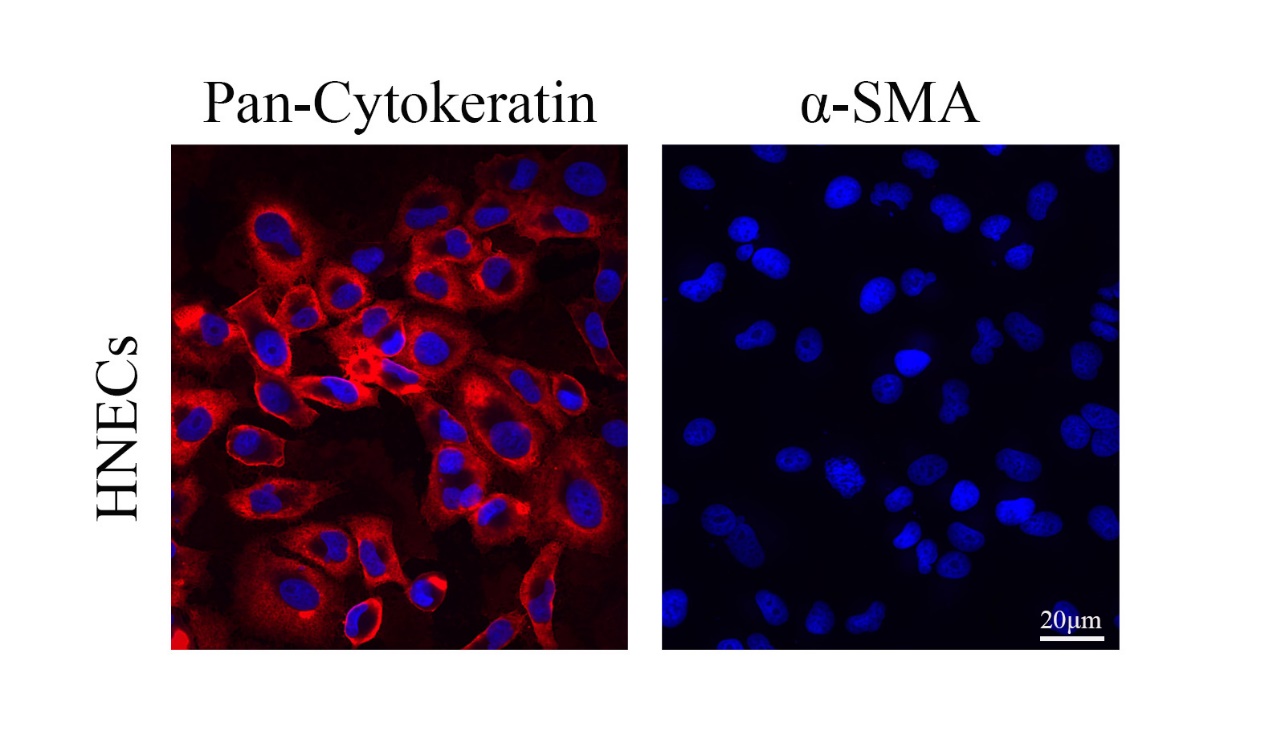

Supplement: Supplementary file 1 [file mmc1.docx]
